# Bathing and toileting difficulties of older adults in rural China: the role of environment

**DOI:** 10.1186/s12877-020-01919-8

**Published:** 2020-12-10

**Authors:** Changxi Liu, Joelle H. Fong, Jasmon W. T. Hoh, Hailin Wu, Yunsheng Dong, Danan Gu, Qiushi Feng

**Affiliations:** 1 Department of Economic Sociology, Shanghai University of Finance and Economics (SUFE), Shanghai, China; 2 Lee Kuan Yew School of Public Policy, National University of Singapore, Singapore, Singapore; 3 Department of Sociology, National University of Singapore, Singapore, Singapore; 4 School of Philosophy and Sociology, Jilin University, Jilin, China; 5 Independent Researcher, New York City, USA; 6 Centre for Family and Population Research (CFPR), National University of Singapore, Singapore, Singapore

**Keywords:** Disability, ADL, Toileting, Bathing, Environment, Older adults, China, Rural area

## Abstract

**Background:**

For older adults, difficulties in bathing and toileting are often the most prevalent in the index of Activities of daily living (ADL). This study aims to examine how environmental factors are associated with difficulty of bathing and toileting among older adults in rural China.

**Method:**

The data are from the 2014 Thousand-Village Survey (TVS), a national survey of Chinese rural residents of old age. The sample consists of 10,689 subjects, 55 years or older, from 536 villages across all provinces of China. Logistic regressions were applied to examine how difficulty of bathing and toileting was related to environmental factors such as geographic location, neighbourhood amenity, and related facilities of bathing and toileting.

**Results:**

Older adults living in the Southern regions of China had lesser difficulty in bathing and toileting than those living in Northern China, controlling for other confounders. Better neighbourhood conditions also reduced the likelihood of having such disabilities. Persons who bathed indoors without showering facilities, in public facilities, and outdoors were significantly more likely to have bathing disability than those who showered indoors with facility. Rural older adults who used pedestal pans and indoor buckets for toileting were more likely to have toileting disability than those who used indoor squatting facilities.

**Conclusion:**

Environmental barriers were associated with functional disability among older adults in rural China, but the disabled individuals may change their environments to adapt to their functional capabilities. Our findings suggest that it is imperative to promote the use of showering facilities and pedestal pans for toileting in rural China.

## Background

Activities of daily living (ADLs) are key measures of functional disability for older adults. The six conventional ADLs include feeding, indoor transferring, clothing, toileting, bathing, and continence [1]. Disability in ADLs is prevalent among older adults and has severe impacts on health and wellbeing [2–5]. Interestingly, across six ADL items, there is a hierarchical structure [1], and difficulties of bathing and toileting often stand out with high prevalence for seniors in daily life [6]. In the UK, for example, the prevalence of bathing and toileting ADL disabilities among older adults aged 75 or older was 34 and 17%, respectively, compared to 12% for dressing and transferring from and/or to bed, and 3% for eating [7]. In mainland China (thereafter China), the incidence rates of bathing and toileting disabilities over a two-year period were 21.6 and 15.8% for oldest-old men and 23.6 and 16.4% for oldest-old women, higher than incidence rates of the other four items [8]. It is reported that 18.2% Chinese oldest-olds, those aged 80 or older , needed assistance in bathing and 8.0% in toileting [9]. And older Chinese adults tend to lose ability to use the toilet independently earlier than their American counterparts [10].

The concerns surrounding difficulties in bathing and toileting among older Chinese adults are exacerbated by rapid population aging in China, currently having around 170 million older adults aged 65 or older [11]. Population aging in China makes it imperative to meet the huge care needs and demands from the large cohorts of Chinese seniors who may subsequently become disabled in later years [12, 13]. Yet, the national insurance of long-term care is still developing, though China recently instituted a national social security system [14, 15]. State-run old-age care institutions have limited capacity, and the private institutions are still underdeveloped [16]. Also worrisome is the decline of traditional role of the family in caring for older adults. Given smaller family sizes, many older Chinese may not have adult children around [17–19].

In contrast to other ADL items, bathing and toileting have special characteristics in the later lives. The onset of bathing and toileting difficulties usually occur earlier in older adults than other ADL limitations [20, 21]. Bathing and toileting are also more demanding activities than the rest of the ADLs because they involve the complex movements of both the upper and the lower body, whereas other activities are mainly restricted to just either upper or lower body movements [7, 8]. In particular, bathing usually involves bending down, turning around, raising of arms and legs, or gripping with fingers, while toileting involves squatting/sitting, standing up as well as actions of hands and arms [22–24]. Both activities also require good capacities of body coordination, balancing and sensory functions [25].

More importantly, both bathing and toileting are embedded in the environment wherein older adults live, and entail interactions between body, facility, infrastructure, climate, temperature, and other environmental factors. These activities are often performed in specialized built environments, namely bathrooms and restrooms, and sometimes involved with the aid of customized facilities [26]. Thus, disabilities in bathing and toileting could occur due to external barriers or lack of aiding facilities, regardless of intrinsic functional limitations. This is in line with the disablement process model, in which both personal and environmental factors could be causes of disability [21]. Along this model, researchers have made efforts to clarify the roles of the intrinsic impairments and external barriers for ADL disability of older adults [27, 28]. In the new classification scheme of WHO, body impairment and disability have been clearly separated with the latter defined with an emphasis on environmental factors [29, 30].

Few studies, however, have examined the role of environment for the two specific ADL limitations in bathing and toileting, although it is well-known that disability in later lives is affected by environmental factors such as geographic location and neighbourhood condition [31–34]. In particular, how related facilities influence these two ADL disabilities, such as the use of pedestal pans for toileting, is important and policy implicative. Such inquiry is also timely given a puzzling pattern revealed by a recent Lancet paper that the declining trend in ADL-based disability in China was simultaneously accompanied by an increasing trend in performance-based functional limitations over 1998–2008 [35]. These seemingly self-conflicting trends may be due to significant improvement of the built environment pertinent to the older population during that period of time, though no follow-up empirical research has been done so far.

This study is to fill this gap in literature. We aim to examine how the environmental factors such as geographic location, neighbourhood condition, and related facility are associated with bathing and toileting difficulties of Chinese older adults. We choose to focus on the rural Chinese in this study, who still have mixed environmental settings in bathing and toileting, unlike their urban counterparts who mostly enjoy standardized modernized environmental settings in these regards [36].

## Method

### Data

The data came from the 2014 Thousand-Village Survey (TVS 2014), which is a national survey of Chinese villages, administered by Shanghai University of Finance and Economics (SUFE). The survey adopted the multi-stage probability proportional to size (PPS) sampling in order for a national representative sample. Based on the national distribution of rural population per the 2010 census, 31 counties were sampled from 21 out of 31 provinces of China (representing about 89% of China’s total rural population). Then, for each county, a town was chosen; for each town, four villages were chosen; in each chosen village, two village groups were finally selected to build up the final sampling frame, from which older adults were randomly sampled. The survey was also implemented by a university-managed fieldwork program, in which rural students of SUFE were asked to obtain the sampling frames of their hometown villages, randomly sample older adults in their hometown villages, and do interviews using questionnaires of the TVS 2014. This program covered all 31 provinces of China. The PPS-based survey and the fieldwork program were conducted concurrently. Older adults who could not communicate properly due to health issues were screened out from interviews. The response rate is around 80%. The final sample used in this analysis contains 10,689 cases of Chinese rural residents aged 55 years or older, from 536 villages across China. Of these, 3698 cases were from the PPS sampling and 6991 from the fieldwork program. More details of this survey are available in the literature [37].

### Variables

The TVS 2014 asked respondents reported whether they had difficulties performing the six ADL activities in daily life within six months at the time of the interview. Our study focused on two specific items of ADLs, namely bathing and toileting, which are most prevalent types of disability among Chinese older adults. These are constructed as separate indicator variables (yes or no).

A number of environmental factors were employed. These included regional-level, neighbourhood-level, and individual-level factors. One variable is geographic location based on the respondent’s province (North, Northeast, East, Central South, Southwest, and Northwest). These regions represented different profiles of temperature and climate as well as socioeconomic development levels in China. To measure the neighbourhood-level environment, we asked the respondents to rate their villages in terms of medical services, old-age care services, sanitation, neighbourhood relations, and security. Each aspect was assessed separately and the response categories include: very satisfied, satisfied, fair, unsatisfied, and very unsatisfied. Following prior studies [38–40], we generated an index of neighbourhood amenity score ranging from 0 to 20 by adding up the five items, with a higher score indicating a better condition of a village.

Detailed measures for facilities of bathing and toileting were also included. These environmental factors accounted for variation at the individual-level in terms of performing these self-care tasks. For bathing, we collected information on how respondents showered on a regular basis. Subjects could select from four different response options including (i) showering indoors, (ii) bathing indoors but no showering, (iii) bathing in public facilities, and (iv) bathing outdoors. Separately, for toileting, the four response options which reflected varied toilet settings included: (i) squatting indoors, (ii) sit-toileting indoors, (iii) using bucket indoors, and (iv) toileting outdoors. These settings reflect the major types of facilities of bathing and toileting in rural China.

We also included a wide set of covariates such as demographic, health, and socioeconomic factors that are associated with ADL disability of older adults as suggested in literature [41–43]. These variables include age, gender, self-reported health (SRH) (bad, fair, or good), having chronical diseases (yes vs. no), economic condition (bad, fair, or good), education (having at least one year of schooling vs. no), occupation (agricultural vs. non-agricultural). The inclusion of SRH is supported by the fact that difficulties in toileting and bathing may be resulted from poor health condition rather than bathing and toileting facilities. We also accounted for respondents’ living arrangements in that coresidence may confound one’s ability to perform bathing and toileting as those living together may provide assistance. Living arrangement has four categories: living alone, living with spouse only, living with children and/or spouse, or living with others.

### Statistical analyses

We applied logistic regression models to investigate how difficulties in bathing and toileting among Chinese older adults were associated with environmental factors. Within each analysis on bathing and toileting, we also further examined gender-age-specific results through dividing respondents by gender (men vs. women) and two age groups (55–74 years old vs. 75 years old or older). STATA 16.0 was used to perform these analyses.

## Results

Table 1 describes the sample characteristics. As shown, out of the 10,689 respondents, 7.4% reported difficulty in bathing, while 4.0% reported difficulty in toileting; for the age group of 75 years old or older, the rates were 16.2 and 7.5%, close to national results of previous literature [9]. The results also show that about 80% Chinese rural seniors bathed indoors at home, among whom half had showering facilities, and about 15% of them used the public bathing rooms. For toileting, about 50% of rural older adults did indoors (30% squatting and 20% sitting), about 40% of them still used toilets outdoors, and very few (about 4–8%) used bucket indoors.

**Table 1:** Description of the sample

	Total	Ages 55–74	Ages 75	Men	Women
Sample size	10,689	8261	2428	5437	5252
Difficulty in Bathing (%)	7.4	4.8	16.2	6.6	8.1
Difficulty in Toileting (%)	4.0	2.9	7.5	3.8	4.1
Bathing facility (%)					
Showering indoors at home	42.4	44.8	34.2	43.0	41.7
Bathing indoors at home	40.3	37.0	51.5	37.9	42.8
Bathing in public facility	14.4	15.4	11.3	15.5	13.4
Bathing outdoors	2.9	2.9	2.9	3.6	2.2
Toileting facility (%)					
Squatting indoors	29.5	29.3	30.2	29.7	29.3
Pedestal pan indoors	24.0	23.5	25.9	23.0	25.1
Bucket indoors	4.4	3.2	8.5	4.2	4.6
Outdoors	42.1	44.1	35.4	43.1	41.1
Neighborhood amenity (mean, SD) (ranges 0–20)	18.8 (3.1)	18.8 (3.1)	18.8 (3.1)	18.8 (3.1)	18.8 (3.1)
Region (%)					
North	10.8	11.8	7.4	10.6	11.0
Northeast	6.0	6.4	4.8	6.0	6.0
East	43.8	42.0	49.8	44.6	43.0
Central South	19.4	19.3	19.7	18.5	20.3
Southwest	15.0	15.0	15.0	15.8	14.2
Northwest	5.0	5.5	3.4	4.6	5.5
Age (mean, SD) (ranges 55–99)	69.0 (7.4)	65.8 (4.5)	79.9 (4.2)	68.9 (7.2)	69.2 (7.6)
Women (%)	49.1	48.6	50.9	0	100
Self-reported health (%)					
Bad	18.6	16.9	24.5	15.4	21.9
Fair	34.5	34.0	36.3	33.4	35.7
Good	46.9	49.1	39.3	51.2	42.4
Having 1 chronic disease (%)	61.5	61.0	63.2	57.7	65.4
Having 1 year schooling (%)	65.6	71.3	46.3	79.6	51.1
Economic condition (%)					
Bad	11.0	10.9	11.2	10.5	11.5
Fair	31.5	31.7	30.8	31.7	31.3
Good	57.5	57.4	58.0	57.9	57.1
Occupation (%)					
Agriculture	80.8	80.3	82.5	73.7	88.2
Non-agriculture	19.2	19.7	17.5	26.3	11.8
Living arrangement (%)					
Live alone	11.7	9.1	20.4	9.9	13.6
Live with spouse only	40.5	43.7	29.9	45.4	35.5
Live with children and/or spouse	45.5	44.9	46.9	42.8	48.1
Live with others	2.4	2.3	2.5	2.0	2.8

Our empirical results reveal strong effects of environment on bathing among older adults (Table 2). Both regional- and neighbourhood-level factors were statistically significant, controlling for other covariates. Relative to those in North, older adults living in the Southern regions of China had less difficulty in bathing. Better neighbourhood conditions were also associated with less bathing problems, but this pattern seems only applicable for women. Individual-level factors were also statistically significant. Compared with showering indoors at home, the three other settings made bathing more challenging for seniors: specifically, bathing indoors without showering, bathing in public facility and bathing outdoors were associated with the increased odds of bathing difficulty by 52% (*p* < 0.001), 46% (*p* < 0.01) and 111% (p < 0.001), respectively. Not surprisingly, this pattern became more obvious for the older age group, i.e. over 75 years old.

**Table 2:** Odds ratios for difficulty in bathing

	Both Sexes			Men			Women		
	Total	Ages 55–74	Ages 75+	Total	Ages 55–74	Ages 75+	Total	Ages 55–74	Ages 75+
Bathing facility									
Bathing indoors at home (showering indoors at home)	1.52*	1.34^$^	1.96*	1.62*	1.43^$^	2.05^#^	1.49^#^	1.34	1.90^#^
Bathing in public facility (showering indoors at home)	1.46^#^	1.05	2.61*	1.55^$^	1.27	2.36^#^	1.48^$^	0.91	3.33*
Bathing outdoors (showering indoors at home)	2.11*	1.35	3.67*	2.45*	1.65	4.23*	1.83	1.08	3.44^$^
Neighborhood amenity [scores 0–20]	0.94*	0.92*	0.96	0.98	0.95	1.01	0.91*	0.89*	0.93^$^
Region									
Northeast (North)	0.99	1.07	0.86	0.82	0.93	0.60	1.14	1.17	1.16
East (North)	0.48*	0.48*	0.47^#^	0.63^$^	0.64	0.53	0.39*	0.37*	0.41^#^
Central South (North)	0.38*	0.34*	0.41*	0.51^#^	0.47^#^	0.50	0.30*	0.26*	0.35^#^
Southwest (North)	0.49*	0.54*	0.40*	0.63^$^	0.66	0.48	0.40*	0.43^#^	0.34^#^
Northwest (North)	1.08	0.80	2.00^$^	1.29	1.15	1.77	0.95	0.59	2.49
Age	1.09*	1.06*	1.10*	1.08*	1.05^#^	1.07^#^	1.11*	1.07*	1.12*
Women (men)	0.89	0.84	1.06	–	–	–	–	–	–
Self-reported health:									
Fair (good)	3.31*	3.57*	3.10*	3.52*	3.90*	3.14*	3.12*	3.19*	3.23*
Bad (good)	8.89*	8.79*	9.53*	9.71*	10.48*	9.34*	8.41*	7.35*	10.71*
Having 1 chronic disease (no)	1.42*	1.34^$^	1.53^#^	1.40^$^	1.16	1.79^#^	1.44^#^	1.60^$^	1.29
Having 1 year schooling (no)	0.79^#^	0.70*	0.98	0.69^#^	0.66^$^	0.72	0.87	0.70^$^	1.39
Economic condition									
Fair (bad)	0.95	0.92	1.03	0.95	0.73	1.74	0.95	1.15	0.66
Good (bad)	0.83	0.72^$^	1.00	0.74	0.57^$^	1.31	0.91	0.87	0.81
Non-agriculture job (agriculture)	1.13	1.22	0.94	1.06	1.13	0.88	1.35	1.48	1.17
Living arrangements									
Live with spouse only (live alone)	1.22	1.10	1.28	1.20	1.06	1.52	1.30	1.18	1.03
Live with children (live alone)	1.90*	1.61^$^	2.13*	1.94*	1.88^$^	2.07^$^	1.89*	1.44	2.19*
Live with others (live alone)	1.32	0.90	1.76	0.82	0.56	1.02	1.80	1.15	2.67^$^
-Log Likelihood	2237.0*	1352.7*	861.3*	1072.2*	666.5*	393.6*	1151.1*	675.3*	453.0*
# of individuals	10,689	8,261	2428	5437	4,245	1,192	5252	4016	1236

Note: The category in the parentheses is the reference group of a variable. ^*^p < 0.001, ^#^*p* < 0.01, ^$^*p* < 0.05

Table 3 further investigated the role of environment in toileting of Chinese rural older adults. Both regional- and neighbourhood-level factors were statistically significant, but the associations were weaker than those for toileting. Specifically, the advantage of Southern China to Northern areas was still existent, and one unit increase in neighbourhood amenity score was associated with the decreased odds of toileting difficulty by about 6% (*p* < 0.01). Interestingly, compared to squatting indoors, toileting outdoors was about 60% more difficult (p < 0.01), the use of pedestal pan indoors about two times more difficult (*p* < 0.001), and use of bucket indoors about 6 times more difficult (p < 0.001). This pattern was consistent across gender and age groups.

**Table 3:** Odds ratios for difficulty in toileting

	Both Sexes			Men			Women		
	Total	Ages 55–74	Ages 75+	Total	Ages 55–74	Ages 75+	Total	Ages 55–74	Ages 75+
Toileting facility									
Pedestal pan indoors (Squatting indoors)	2.67*	3.00*	2.23^#^	3.75*	6.03*	2.04	1.95^#^	1.62	2.63^$^
Bucket indoors (Squatting indoors)	6.69*	5.95*	7.38*	8.26*	9.87*	6.65*	6.05*	4.43*	9.75*
Outdoors (Squatting indoors)	1.60^#^	1.57^$^	1.69^$^	1.48	1.76	1.29	1.79^#^	1.53	2.44^$^
Neighborhood amenity [scores 0–20]	0.94^#^	0.92^#^	0.98	0.95	0.91^#^	1.02	0.93^#^	0.93^$^	0.94
Region									
Northeast (North)	1.23	1.22	1.27	1.16	1.67	0.42	1.30	0.89	2.86
East (North)	0.43*	0.41*	0.49^$^	0.42^#^	0.43^$^	0.36^$^	0.42^#^	0.36^#^	0.67
Central South (North)	0.58^#^	0.50^#^	0.73	0.47^$^	0.34^#^	0.36	0.67	0.59	0.97
Southwest (North)	0.84	0.90	0.74	1.05	1.10	0.83	0.65	0.70	0.65
Northwest (North)	0.64	0.61	0.66	0.64	0.75	0.56	0.62	0.52	0.81
Age	1.06*	1.05^#^	1.10*	1.05*	1.04	1.07	1.07*	1.06^#^	1.13*
Women (men)	0.83	0.86	0.78	–	–	–	–	–	–
Self-reported health:									
Fair (good)	2.99*	2.98*	3.10^#^	3.64*	3.89*	3.57^#^	2.34^#^	2.17^$^	2.77^$^
Bad (good)	11.37*	10.65*	13.35*	16.15*	17.59*	15.94*	7.81*	6.58*	11.88*
Having 1 chronic disease (no)	1.01	0.91	1.28	1.09	0.83	1.66	0.98	0.99	1.02
Having 1 year schooling (no)	0.78^$^	0.76	0.81	0.75	0.83	0.65	0.82	0.72	1.17
Economic condition									
Fair (bad)	0.75^$^	0.89	0.55^#^	0.78	0.67	0.93	0.73	1.16	0.31*
Good (bad)	0.59*	0.59^#^	0.54^#^	0.57^#^	0.51^$^	0.67	0.60^$^	0.70	0.43^$^
Non-agriculture job (agriculture)	1.42^$^	1.45^$^	1.34	1.34	1.30	1.36	1.59	1.75	1.20
Living arrangements									
Live with spouse only (live alone)	1.47^$^	1.21	1.59	1.26	0.92	1.78	1.60	1.54	0.77
Live with children (live alone)	1.80*	1.36	2.33*	1.48	1.30	1.55	2.07*	1.44	3.00*
Live with others (live alone)	1.34	1.19	1.34	0.45	0.36	0.49	2.37^$^	1.95	3.22
-Log Likelihood	1416.4*	915.2*	490.5*	674.6*	431.0*	231.9*	729.7*	469.4*	242.0*
# of individuals	10,689	8,261	2428	5437	4245	1192	5252	4016	1236

Note: The category in the parentheses is the reference group of a variable. *p < 0.001, #*p* < 0.01, $*p* < 0.05

The results of covariates are also noteworthy. As expected, younger age, better SRH, and better socioeconomic status were associated with less difficulty of rural older adults in bathing and toileting. Interestingly, living with children was significantly related to bathing and toileting difficulty in later year, as deserves discussion later.

## Discussion

For disability in later years, difficulties in bathing and toileting often have high prevalence rates among older adults. Based on the data from the TVS 2014, this study investigated how difficulties of bathing and toileting of Chinese rural seniors are affected by environmental factors such as geographic location, neighbourhood condition and related facility. We found that environment plays an important role in bathing and toileting in later years, whereas the association of environment on disability is not unidirectional, but interactive. The disadvantaged environmental condition was highly related to more bathing difficulties of older adults; yet people with functional limitations tend to adjust environment to facilitate their toileting.

We found significant regional disparities in bathing and toileting limitations among older adults in rural China. This result is consistent with previous studies which have highlighted that ADL disabilities tend to be more prevalent in the Northern region [34]. Since a substantial proportion of older adults in rural China go outdoors for bathing (about 15%) and toileting (about 40%), the climate directly influences their ability to perform these tasks. Another possible explanation for the observed regional patterns is the uneven economic development in China. Eastern and Southern provinces in China usually have a better socioeconomic development level than their Northern and Western counterparts [44], and older adults in affluent regions may have better resources to cope with barriers of daily life. More research is clearly needed to shed light on this topic.

This study also confirms that the important role of neighbourhood factors in explaining differences in bathing and toileting disabilities. A number of studies have found that neighbourhood conditions such as lighting, good traffic, security, sanitation, and public services etc. are positively associated with health and wellbeing of older residents [45–47]. Along this line of work, our study further demonstrated that good neighbourhood amenities were also related to less difficulty in bathing and toileting among Chinese rural older adults, many of whom still rely on non-home based or public facilities for bathing and toileting.

Importantly, we examined in detail the facilities of bathing and toileting for older adults, an aspect that is largely omitted in the existing literature. Unlike seniors from developed societies, rural Chinese older adults have various manners of bathing and toileting. For bathing, showering indoors at home as the modern and convenient way of bathing was only adopted by about 40% of rural seniors in the early 2010s. As expected, older people using the other bathing methods encountered more difficulties. They may have to carry additional tasks such as preparing hot water and visiting a place beyond home, deal with unexpected contingencies, and suffer a situation without surveillance of family members [25, 48].

Patterns of toileting use is rather intriguing among older Chinese adults in rural areas. A substantial proportion of these older adults (about 40%) still reported doing toileting outdoors rather than indoors, and about 30% of the sample reported doing toileting by squatting indoors. The remainder, who used buckets indoors and pedestal pans indoors for toileting, reported the most difficulty in toileting. This is somewhat counterintuitive since buckets and pedestal pans are supposed to make toileting easier. We believe one possible explanation could be that when squatting becomes harder for frail older adults, devices such as pedestal pan seats and bucket seat toilets are often installed to mitigate the issue and lower the risk of falls [49, 50]. This is consistent with the environmental proactivity hypothesis, that is, older persons with illness tend to make changes to their environments [51, 52], though our cross-sectional data could not directly test this. While some changes could be due to active adaptation, some could be involuntary when the health decline in old age limits the choice of older adults for toileting. There is additional evidence for this explanation. For example, we observed that the use of buckets and pedestal pans for toileting indeed increased with age (see Table 1). Additionally, compared with solo-living older adults in rural China, those who lived with their adult children were much more likely to experience difficulties in bathing and toileting (see Table 3). Similar to the logic of the environmental proactivity hypothesis, Chinese rural older adults with health declines often chose to live with children for help, a norm of old-age care in China [53, 54]. Lastly, why does this interesting scenario not happen for bathing? A partial reason, we think, is that many older adults in rural China may avoid bathing if health status disallows, instead of actively seeking for countermeasures.

Our findings in this paper confirmed the significant impact of environment on old-age disability, and thus provided good support for the speculations on the puzzling disability trends of Chinese older adults, as raised by Zeng and colleagues (2017). That is, improvement in facilities and infrastructures of the older adult living environments could effectively reduce their disability, even if their intrinsic functional limitations become worsened [35]. Such a conclusion has important policy implications for societies of population aging. That is, even though the population aging could lead to a decline in general population health, there could be room to reverse this negative trend through modifying environment towards more age-friendly designs and solutions. For the Chinese rural older adults specifically, this study highlighted the urgencies to promote the use of showering facilities and pedestal pans for toileting.

The importance of the role of environment in the later life, as shown in this study, is in line with a recent review on mechanism and framework of disability that highlights the role of environment in associating disability among older adults [55]. And it also matches in line with the recent framework of healthy aging as proposed by WHO [56]. In this new framework, capacity and ability of older adults are clearly separated so that intrinsic capacity defined as the composite of all physical and mental capacities of an individual is considered only a part of functional ability for older adults, for which “the environments they inhabit and their interaction with them are also major determinants of what older people can do” (p.2149). Therefore, healthy aging is essentially about interactions of older adults and their environment. Our study is aligned with this general direction as we have demonstrated how environment matters for bathing and toileting, the two major types of disability in older adults.

This study also reminds us about the complicacy of the disablement process, which is not a purely biomedical issue, but heavily affected by external barriers and facilitators. One direct implication is thus to put caution on the comparability of the routine disability measures such as ADL across nations. Societies with different developmental stages often have diversified contexts for older adults to perform daily activities. Direct comparisons could be misleading, as the results are often mixed with both intrinsic body limitations and external factors. For efforts to clarify these intertwining relations, as suggested by our results, it is also needed to go beyond regional- and neighbourhood-level factors to pay attentions to related facilities.

There are a number of limitations in this study. Our data are cross-sectional, and thus findings of this study on the effect of environmental factors are associational rather than causal. The data was also about 6 years old in the year of 2020. Although rural China has witnessed noticeable changes within this period of time, the general situation in these areas, especially those in Central and Western China, has not been substantially altered [36]. In addition, the study excluded those who could not communicate properly due to health issues, which constrained us from understanding the needs of severely unhealthy older adults, especially those with cognitive impairment. We thus encourage future studies to collect better data to address these limitations. In term of measurements, while we classified a few major types of facilities of toileting and bathing for older adults in rural China, there is surely room of improvement for further research to refine these important measurements. The neighbourhood amenity score is also not a validated index, though with a good face validity by covering major old-age-related characteristics of Chinese rural neighbourhood. We thus expect the development of a good measurement scheme for the neighbourhood environment of older adults in China. Lastly, a part of the sample of the TVS 2014 did not come from national PPS sampling, but from the fieldwork of the SUFE students of the rural areas. However, these students came from all of China with quite even chance of being admitted to SUFE, and we thus perceived the sampling bias not severe.

## Conclusion

Through examining the environmental factors of bathing and toileting among older adults in rural China, we found geographic location, community condition and related facilities were significantly associated with difficulties of bathing and toileting in the later life. Such associations seem not unidirectional, but interactive. This study suggests policy interventions to improve the environment of daily lives of Chinese rural seniors, especially related facilitiesof bathing and toileting, which may effectively reduce their disability in later years.

## Data Availability

The data that support the findings of this study is available from SUFE, but restrictions apply to the availability of these data, which were used under license for the current study, and so are not publicly available. Data are however available from the authors upon reasonable request and with permission of SUFE.

## Abbreviations

ADL: Activities of daily living
SRH: Self-reported health
TVS: Thousand-Village Survey
PPS: Probability proportional to size
SUFE: Shanghai University of Finance and Economics
